# Discovery of a preliminary urinary metabolite panel for Parkinson’s disease: a pilot study using paired patient-spouse samples and machine learning consensus

**DOI:** 10.3389/fneur.2026.1763253

**Published:** 2026-06-09

**Authors:** Qian-Qian Chen, De-Hai Gou, Jin-Yu Huang, Zhen-Hua Mo, Xiao-Yong Guan, Jia-Ning Xu

**Affiliations:** 1 Faculty of Medicine, Guangxi University of Science and Technology, Liuzhou, Guangxi, China; 2 Guangzhou National Laboratory, Guangzhou International Bio Island, Guangzhou, Guangdong, China; 3 The First Affiliated Hospital of Guangxi University of Science and Technology, Guangxi University of Science and Technology, Liuzhou, China; 4 School of Electronic Engineering, Guangxi University of Science and Technology, Liuzhou, Guangxi, China

**Keywords:** machine learning, non-invasive diagnostic biomarkers, Parkinson’s disease, partial least squares discriminant analysis, random forest, support vector machine, urine

## Abstract

**Introduction:**

Parkinson’s disease (PD) lacks reliable non-invasive diagnostic biomarkers. Urine is a promising biofluid for biomarker discovery, but the profound influence of shared environment and lifestyle represents a major confounder.

**Methods:**

To rigorously address this, we designed a pilot study using a unique matched-pair cohort: PD patients together with their healthy spouses. Untargeted LC–MS metabolomics was performed on urine samples from 15 carefully matched pairs. Differential features were identified using VIP > 1.0 and *p* < 0.05. A multi-model consensus strategy (RF, SVM, PLS-DA) was applied to prioritize robust candidates from 2,640 annotated metabolites, followed by filtering for pharmacological relevance and collinearity.

**Results:**

A preliminary five-metabolite panel (Cyanuric acid, Benzeneacetonitrile, 3-Formylsalicylic Acid, dADP, and ent-cassa-12,15-dien-2beta-ol) was defined. Despite the inherently small sample size of this niche cohort, the panel demonstrated promising internal discriminative performance (AUC > 0.95).

**Discussion:**

We emphasize that these results are exploratory. The primary contribution of this pilot study is not a validated diagnostic tool, but the demonstration of a carefully controlled design to isolate PD-specific metabolic signatures and the proposal of specific candidate biomarkers. This work establishes a critical proof-of-concept and prioritizes targets for essential future validation in larger, independent cohorts.

## 1 Introduction

Parkinson’s disease (PD) is the second most common neurodegenerative disorder worldwide, affecting approximately 1% of the population over the age of 60 (1). It is characterized by progressive motor and non-motor symptoms (2), and its early diagnosis remains a substantial clinical challenge (3). There is an urgent need for reliable, non-invasive biomarkers to assist in this process (4).

Untargeted metabolomics, which enables unbiased profiling of metabolites, holds great promise for discovering such disease-associated alterations (5, 6). Urine is a particularly attractive biofluid for this purpose due to its non-invasive collection and rich metabolic content (7). However, a major hurdle in urinary metabolomic studies of chronic diseases like PD is the substantial confounding effect of lifelong environmental and lifestyle factors, which can obscure true disease-specific signals.

To address this fundamental challenge, we designed the present pilot study to leverage a unique and rigorous approach: the analysis of PD patients together with their healthy spouses as matched controls. This patient-spouse paired design is exceptionally useful for controlling shared environmental exposures, diet, and socioeconomic factors over decades. Nevertheless, constructing such a cohort is inherently difficult due to the scarcity of elderly couples where one partner has PD and the other is a fully eligible healthy control, inevitably limiting the achievable sample size. We posit that this trade-off—a smaller but exquisitely controlled sample—is justified for an exploratory investigation aiming to isolate relatively stable metabolic signatures with minimal confounding.

Within this framework, we employed untargeted LC–MS metabolomics and applied a multi-model machine learning consensus strategy (integrating RF, SVM, and PLS-DA) to prioritize robust candidate biomarkers from this unique cohort. The primary aim of this pilot study was not to define a validated diagnostic tool, but to utilize this stringent design as a discovery platform to identify a preliminary set of urinary metabolite candidates that warrant prioritization in future large-scale validation studies.

## 2 Materials and methods

### 2.1 Study design and participants

This study was conducted using a matched case–control design. All participants were recruited from the First Affiliated Hospital of Guangxi University of Science and Technology in Liuzhou, Guangxi, China. The case group included patients clinically diagnosed with PD according to the Movement Disorder Society (MDS) Clinical Diagnostic Criteria for Parkinson’s Disease (2015), an internationally recognized and widely accepted standard. For each PD patient, a healthy spouse was recruited as a matched control to minimize the influence of shared environmental and lifestyle factors.

All participants were long-term residents of Liuzhou and were presumed to share similar dietary habits and environmental exposures. First-morning urine samples were collected from all participants using sterile centrifuge tubes. The samples were immediately aliquoted, flash-frozen in liquid nitrogen for 15 min, and stored at - 80 °C until metabolomic analysis.

Ethical approval for this study was obtained from the Institutional Ethics Committee of Guangxi University of Science and Technology. All procedures adhered to the principles of the Declaration of Helsinki, and written informed consent was obtained from all participants.

### 2.2 Sample collection and preparation

#### 2.2.1 Urine sample collection

Midstream morning urine samples were collected from all participants using sterile centrifuge tubes under standardized conditions. Each sample was immediately aliquoted into two sterile 500 μL tubes, flash-frozen in liquid nitrogen for 15 min, and stored at −80 °C. To preserve metabolite integrity, all samples were transported on dry ice and maintained at −80 °C throughout handling and storage. All urine samples were collected as first-morning void before the patient’s first daily medication to minimize acute drug effects. However, no drug washout was performed for ethical and clinical reasons.

#### 2.2.2 Metabolite extraction

For liquid chromatography–mass spectrometry (LC–MS) analysis, frozen urine aliquots were thawed on ice (4 °C) and vortexed for 1 min to ensure homogeneity. A defined volume of each sample was transferred to a 2 mL centrifuge tube, followed by the addition of 100 μL of 2-chloro-L-phenylalanine (4 ppm in 80% methanol, CAS, 103616-89-3, Aladdin) as an internal standard. The mixture was vortexed for 1 min and centrifuged at 12,000 rpm for 10 min at 4 °C (H1850-R centrifuge, Xiangyi, China). The supernatant was filtered through a 0.22 μm PTFE membrane (Jinteng, China) and transferred to LC–MS vials for analysis.

#### 2.2.3 Quality control

A pooled quality Control (QC) sample was generated by combining equal volumes of all 30 extracted samples to monitor analytical stability and correct for instrument-derived variations.

### 2.3 LC–MS analysis

Untargeted metabolomic profiling was performed using a Thermo Vanquish UHPLC system coupled with a Thermo Orbitrap Exploris 120 mass spectrometer (Thermo Fisher Scientific, USA). Chromatographic separation was achieved on an ACQUITY UPLC® HSS T3 column (2.1 × 100 mm, 1.8 μm; Waters, USA) maintained at 40 °C, with a flow rate of 0.3 mL/min and injection volume of 2 μL. The mobile phase compositions were: Positive ion mode: Aqueous phase (A2): 0.1% formic acid in water (v/v); Organic phase (B2): 0.1% formic acid in acetonitrile (v/v). Negative ion mode: Aqueous phase (A3): 5 mM ammonium formate in water; Organic phase (B3): Acetonitrile. Gradient program for both modes: 0–1 min: 10% B; 1–5 min: 10% → 98% B; 5–6.5 min: 98% B; 6.5–6.6 min: 98% → 10% B; 6.6–8 min: 10% B.

Mass spectrometry utilized electrospray ionization with spray voltages of +3.50 kV (positive) and −2.50 kV (negative), sheath gas (40 arb), auxiliary gas (10 arb), and capillary temperature (325 °C). Full-scan MS1 spectra (resolution: 60,000; *m/z* 100–1,000) were acquired, followed by data-dependent MS^2^ (ddMS^2^) scans (resolution: 15,000; normalized collision energy: 30%) targeting the top 4 ions with dynamic exclusion.

### 2.4 Data preprocessing and multivariate analysis

#### 2.4.1 Data preprocessing

Format conversion: Raw mass spectrometry files (.raw format) were converted to the mzXML format using the MSConvert tool from the ProteoWizard software suite (v3.0.8789) to enable downstream processing.

Peak processing: Peak detection, retention time alignment, and integration were conducted in R using the XCMS package (v3.12.0). The centWave algorithm was employed with the following parameter settings: bw = 2: bandwidth for peak grouping; ppm = 15: mass accuracy tolerance; peakwidth = *c*(5, 30): expected minimum and maximum chromatographic peak width (in seconds); mzwid = 0.015: m/z bin width; mzdiff = 0.01: minimum m/z difference for resolving overlapping peaks.

#### 2.4.2 Technical quality assessment

Systematic technical variation was corrected using support vector regression normalization, based on pooled QC samples distributed throughout the analytical sequence. Metabolic features with a coefficient of variation (CV) exceeding 30% across QC replicates were removed to ensure data quality and reproducibility.

Technical quality assessment. Principal component analysis (PCA) was performed using the ropls package (v1.22.0) in R to evaluate data structure and technical consistency. The data matrix was autoscaled (mean-centered and scaled to unit variance) prior to analysis.

Acceptance criteria. Tight clustering of QC samples within the 95% confidence ellipse on PCA score plots. At least 65% of all detected features exhibiting CV < 30% across QC injections.

#### 2.4.3 Supervised multivariate modeling

Orthogonal projections to latent structures discriminant analysis (OPLS-DA) was applied to maximize discrimination between PD patients and healthy spousal controls (NC). This supervised method partitions metabolic variance into: Predictive components: capturing group-related differences; Orthogonal components: capturing structured noise unrelated to group separation. Model Performance Parameters: *R*^2^*X* (cum): cumulative variance explained in the predictor (*X*) matrix; *R*^2^*Y* (cum): cumulative variance explained in the response (*Y*) matrix; *Q*^2^ (cum): cross-validated predictive ability, estimated via 7-fold cross-validation. Model Validation: Model robustness was assessed through 100-fold permutation testing. Criteria for a valid model included: The original *Q*^2^ value exceeding all permuted *Q*^2^ values; A negative intercept in the regression line of permuted *Q*^2^ values against model correlation.

#### 2.4.4 Univariate statistical analysis and multiple testing correction

Differential metabolites between PD patients and NC were initially screened using univariate statistical analysis combined with supervised multivariate metrics. Univariate statistical significance was initially evaluated using Student’s t-test with Benjamini–Hochberg FDR correction. Separately, VIP scores derived from OPLS-DA were used as an auxiliary multivariate prioritization metric. Features meeting the exploratory screening criteria of raw *p* < 0.05 together with VIP > 1.0 were retained for downstream exploratory analysis.

Although participants were recruited using a matched patient–spouse design to minimize long-term environmental confounding, differential metabolite screening was performed using unpaired Student’s t-tests because the primary objective of this pilot study was exploratory feature discovery and global feature prioritization rather than estimation of within-pair effect sizes.

Fold-change values were evaluated descriptively during downstream interpretation; however, no predefined fold-change threshold was applied during feature screening in this exploratory metabolomics study, in order to avoid excluding potentially informative metabolites with moderate abundance changes but strong statistical or classification relevance. This permissive strategy was intentionally adopted for exploratory biomarker discovery in this pilot cohort and was followed by multiple downstream filtering and consensus-selection procedures to reduce false-positive prioritization.

### 2.5 Annotated metabolite analysis and biomarker discovery

#### 2.5.1 Metabolite annotation

Annotation Strategy: Putative identification was performed by matching experimental m/z values and MS/MS spectra to the following: Public databases: HMDB, KEGG, LipidMaps, MassBank, mzCloud; In-house library: Panomix Biomedical’s proprietary metabolite standard database; Identification Criteria: Mass accuracy threshold: within ±30 ppm; MS/MS validation: comparison of experimental and reference fragment ion spectra.

#### 2.5.2 Machine learning-based biomarker selection

To identify exploratory candidate biomarkers, the processed secondary metabolite dataset was analyzed using three machine learning algorithms—RF, SVM, and PLS-DA—implemented in MetaboAnalyst 5.0, following a customized multi-model screening protocol.

##### 2.5.2.1 RF analysis

RF classification was performed using MetaboAnalyst’s RF module with customized parameters. To ensure stability, ten independent RF models were trained using different random seeds. Each model constructed 500 decision trees and ranked features based on Mean Decrease Accuracy. For each iteration, the top 20 ranked metabolites were selected. The intersection of the top 20 metabolites across all 10 RF runs was defined as the RF-consensus feature set, representing consistently high-ranking variables.

##### 2.5.2.2 SVM analysis

SVM analysis was conducted using a linear kernel with recursive feature elimination (RFE) to optimize variable selection. Features were ranked based on the magnitude of their weight coefficients in the final model, and the top 20 metabolites were selected for cross-comparison.

##### 2.5.2.3 PLS-DA

PLS-DA was applied via MetaboAnalyst’s built-in module. Variable importance in projection (VIP) scores were computed to estimate each metabolite’s contribution to class separation. Features were ranked according to their VIP values, and the top 20 ranked metabolites were retained.

##### 2.5.2.4 Consensus biomarker selection

Final candidate biomarkers were defined as the intersection of the three feature selection results, including: Metabolites present in the RF-consensus set (i.e., appearing in the top 20 across all 10 RF iterations); The top 20 SVM-RFE-selected features, and the top 20 VIP-ranked features from PLS-DA.

This multi-model consensus strategy was adopted to enhance the robustness of candidate prioritization under exploratory conditions. However, we acknowledge that consensus selection within the same dataset cannot fully eliminate the risk of overfitting and therefore requires future external validation.

### 2.6 Biomarker evaluation

#### 2.6.1 Single feature-based ROC analysis

To evaluate the discriminative ability of individual candidate metabolites, boxplots were generated to visualize the distribution differences between PD patients and matched NC. These plots clearly illustrate the group differences and potential outliers.

In addition, a combined receiver operating characteristic (ROC) curve was constructed based on the aggregated prediction probabilities from all candidate metabolites to assess the overall classification performance when these single features are considered jointly. The area under the curve (AUC) of the combined ROC quantifies the overall diagnostic accuracy.

#### 2.6.2 Multivariate model performance evaluation

##### 2.6.2.1 Variable filtering prior to modeling

To improve model stability and biological interpretability, a systematic variable selection process was applied prior to model evaluation. The following criteria were used to filter candidate metabolites:

Pharmacological relevance to PD Metabolites. Directly related to PD medications—such as dopamine, L-DOPA precursors, or compounds influenced by dopaminergic pathways—were prioritized for exclusion, as they may reflect treatment effects rather than disease-specific alterations.

Collinearity assessment (Variance Inflation Factor, VIF). To minimize multicollinearity and avoid overfitting, variables with excessive multicollinearity (typically VIF > 10) were evaluated iteratively in combination with biological relevance and pairwise correlation structure. This ensured that retained features contributed independent predictive information.

Pairwise correlation analysis (Pearson’s *r*). Highly redundant metabolites exhibiting strong pairwise correlations (*r* > 0.9) were excluded. Among correlated pairs, the variable with higher biological relevance or statistical importance was retained.

This filtering strategy was designed to eliminate non-specific, unstable, or redundant variables, thereby enhancing the robustness of downstream biomarker modeling.

##### 2.6.2.2 Multivariate model performance evaluation

To evaluate the predictive performance of the selected metabolite panel, a repeated pair-aware 5-fold cross-validation (CV) procedure was implemented using Logistic Regression (LR) and SVM classifiers with specified model parameters. The detailed methodology is described below:

Data preprocessing and feature selection. Candidate metabolite features selected from prior analyses were extracted. Zero values were replaced by half of the minimum non-zero value per feature to avoid issues during log transformation. Samples were normalized by dividing each row by its sum, followed by natural log transformation (log1p) to stabilize variance.

Pair identification and grouping. Each sample was assigned a pair ID derived from its identifier to represent matched PD patient and healthy spouse pairs. This pair ID was used as the grouping variable in cross-validation to ensure that samples from the same pair were always assigned to the same fold.

Repeated pair-aware 5-fold cross-validation. Cross-validation splits were generated using scikit-learn’s GroupKFold with groups = pair_ids to guarantee that paired samples were kept together within each fold, preventing information leakage. The 5-fold CV was repeated 10 times with fixed random seeds to improve reliability.

Within each fold, a StandardScaler was fit only on the training data to standardize features (zero mean, unit variance) and then applied to the test data, avoiding data leakage.

Logistic Regression was configured with a maximum iteration limit of 1,000 to ensure convergence, while SVM was configured with probability = True to enable probability output for ROC analysis.

Model training and evaluation. Models were trained on standardized training folds and evaluated on the corresponding test folds. Predictions generated probabilities for the positive class. Performance metrics including ROC AUC and classification accuracy were calculated per fold.

Aggregation of performance metrics. Final performance metrics were derived by aggregating predictions across all 50 test folds (10 repeats × 5 folds). This approach provides an overall estimate of model discriminative ability and variability. Because feature prioritization was performed prior to classifier evaluation, the resulting performance estimates should be interpreted as exploratory internal validation rather than definitive generalizable predictive performance. Therefore, classifier performance estimates may retain optimistic bias despite pair-aware cross-validation and hold-out evaluation.

Internal hold-out validation and permutation testing. An internal hold-out validation was performed by stratified splitting of pairs into training (70%) and testing (30%) sets. Models were trained and tested similarly with feature standardization fit on the training set.

Permutation testing with 1,000 iterations on the hold-out set was conducted to assess the statistical significance of observed AUC values.

## 3 Results

### 3.1 Participant characteristics

Demographic and clinical characteristics of the 15 PD patients and their 15 spousal controls are summarized in Table 1. The mean age of PD patients was 66.31 ± 7.25 years, while that of controls was 63.00 ± 9.37 years; the difference was not statistically significant (*p* = 0.139). Body mass index (BMI) was also comparable between groups (PD: 22.49 ± 3.51 kg/m^2^; Control: 21.64 ± 2.85 kg/m^2^; *p* = 0.419). Consistent with the matched-pair design, gender distribution showed male predominance in PD patients (3 females/12 males) versus female predominance in controls (12 females/3 males), though this difference did not reach statistical significance by Fisher’s exact test (*p* = 0.07).

**Table 1:** Demographic and clinical characteristics of PD patients and NC.

Variable	PD patients (*n* = 15)	NC (*n* = 15)	*p*-value
Age (years, mean ± SD)	66.31 ± 7.25	63.00 ± 9.37	0.139
Sex (female/male)	3/12	12/3	0.07
BMI (kg/m^2^, mean ± SD)	22.49 ± 3.51	21.64 ± 2.85	0.419
Disease duration, years†	6.0 (3.3–8.8)	–	–
UPDRS-III score†	40.0 (33.5–45.8)	–	–
Hoehn and Yahr stage†	2.5 (1.0–3.0)	–	–
Levodopa-containing therapy, *n* (%)†	12 (85.7%)	–	–
Dopamine agonist use, *n* (%)†	10 (71.4%)	–	–
MAO-B inhibitor use, *n* (%)†	1 (7.1%)	–	–
Anticholinergic use, *n* (%)†	1 (7.1%)	–	–

Data are presented as mean ± SD, median (interquartile range), or *n* (%). BMI: body mass index; UPDRS-III: Unified Parkinson’s Disease Rating Scale Part III. Levodopa-containing therapy included Madopar, Sinemet, and levodopa/benserazide formulations. Dopamine agonists included piribedil and pramipexole. MAO-B inhibitors included selegiline and rasagiline. ^†^Complete clinical severity and medication information were available for 14 of the 15 PD patients due to incomplete clinical records in one case.

A total of 30 urine samples (15 matched pairs) were collected under standardized protocols. All samples met pre-analytical QC requirements, including proper handling, immediate freezing, and storage at −80 °C. Pooled QC samples were prepared and included throughout the analytical sequence to monitor technical variability during LC–MS acquisition.

All urine samples were collected, processed, and stored following standardized protocols to preserve metabolite integrity. Pooled QC samples were prepared by combining equal volumes of all extracted samples for subsequent system stability assessment during LC–MS acquisition.

### 3.2 Data quality and technical assessment

To evaluate the stability and reproducibility of the LC–MS-based untargeted metabolomics platform, pooled QC samples were systematically injected throughout the analytical sequence. These QC samples, prepared by combining equal volumes of all study samples, served as internal references to monitor system performance and ensure data reliability.

**Figure 1:**
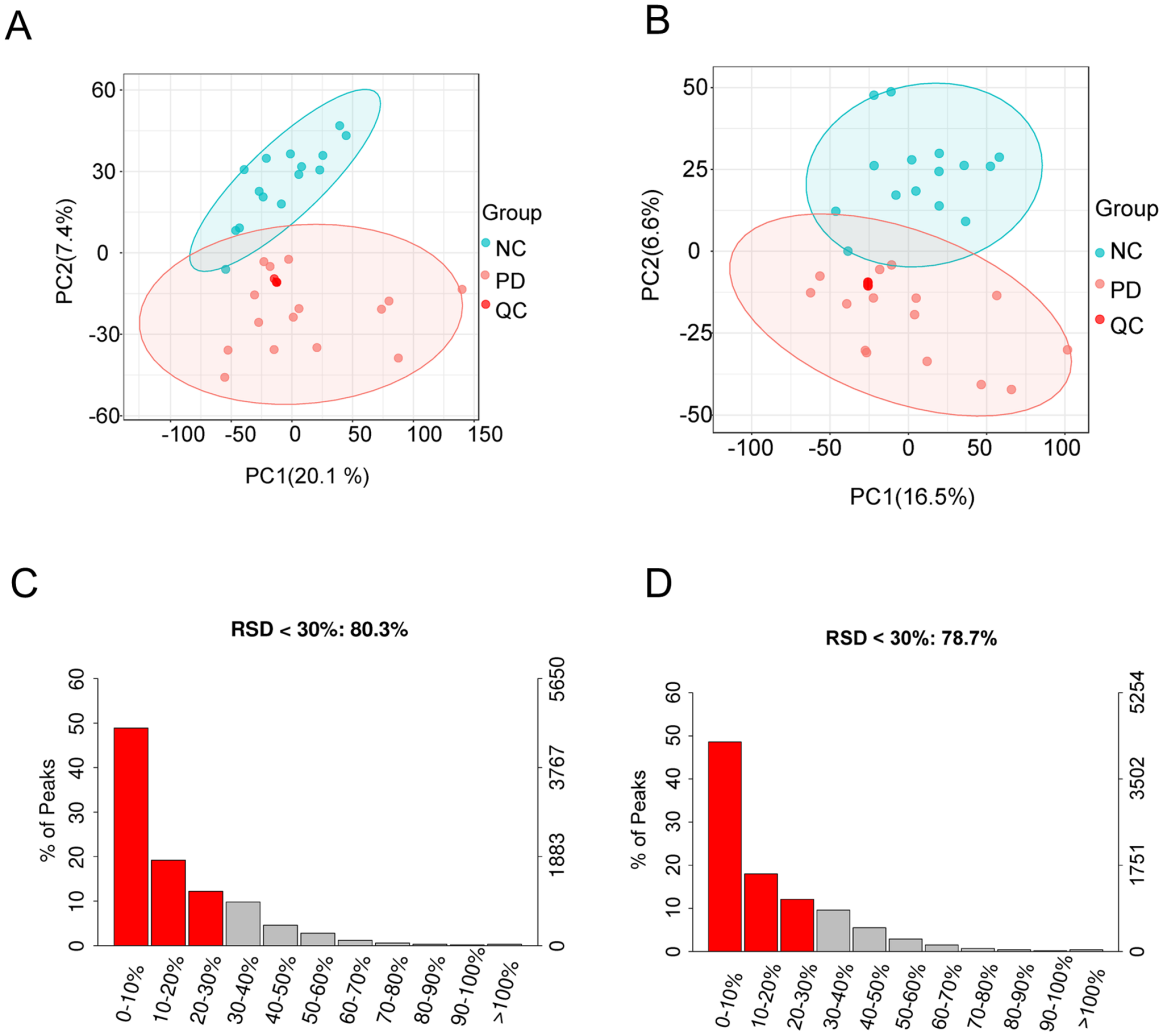
Technical quality assessment of LC–MS-based metabolomics data. **(A, B)** PCA score plots of all urine samples, including Parkinson’s disease (PD) patients, NC, and pooled QC samples, based on metabolic profiles in the positive ion mode **(A)** and negative ion mode **(B)**. QC samples (red dots) clustered tightly within the 95% confidence ellipses, indicating good analytical stability and minimal batch effects. **(C, D)** Distribution of relative standard deviation (RSD) values across features in QC samples for the positive ion mode **(C)** and negative ion mode **(D)**. Features with RSD < 30% accounted for 80.3 and 78.7% of the total features in the positive and negative modes, respectively, indicating high technical reproducibility.

PCA was first performed to visualize sample clustering and detect potential batch effects. As shown in Figures 1A,B, QC samples (red dots) formed tight clusters within the 95% confidence ellipses in both the positive (Figure 1A) and negative ion modes (Figure 1B), indicating high instrumental stability and consistent data acquisition. The separation between PD patients and NC was also evident, suggesting potential biological variation suitable for downstream analysis.

In addition to PCA-based assessment, the CV, expressed as relative standard deviation (RSD), was calculated for all metabolic features across QC replicates. Features with RSD > 30% were considered analytically unstable and excluded from further analysis. As shown in Figures 1C,D, a high proportion of features demonstrated strong reproducibility, with 80.3 and 78.7% of features retained in the positive and negative modes, respectively, after RSD filtering.

Together, these results demonstrate that the metabolomics dataset met rigorous quality control criteria, ensuring that the retained metabolic features are technically relatively stable and suitable for subsequent multivariate modeling and biomarker discovery.

### 3.3 Metabolic profiling and group discrimination

Orthogonal partial least squares-discriminant analysis (OPLS-DA) was performed to assess metabolic differences between PD patients and matched NC. Clear group separation was observed in both positive and negative ion modes, indicating distinct metabolic alterations associated with PD.

**Figure 2:**
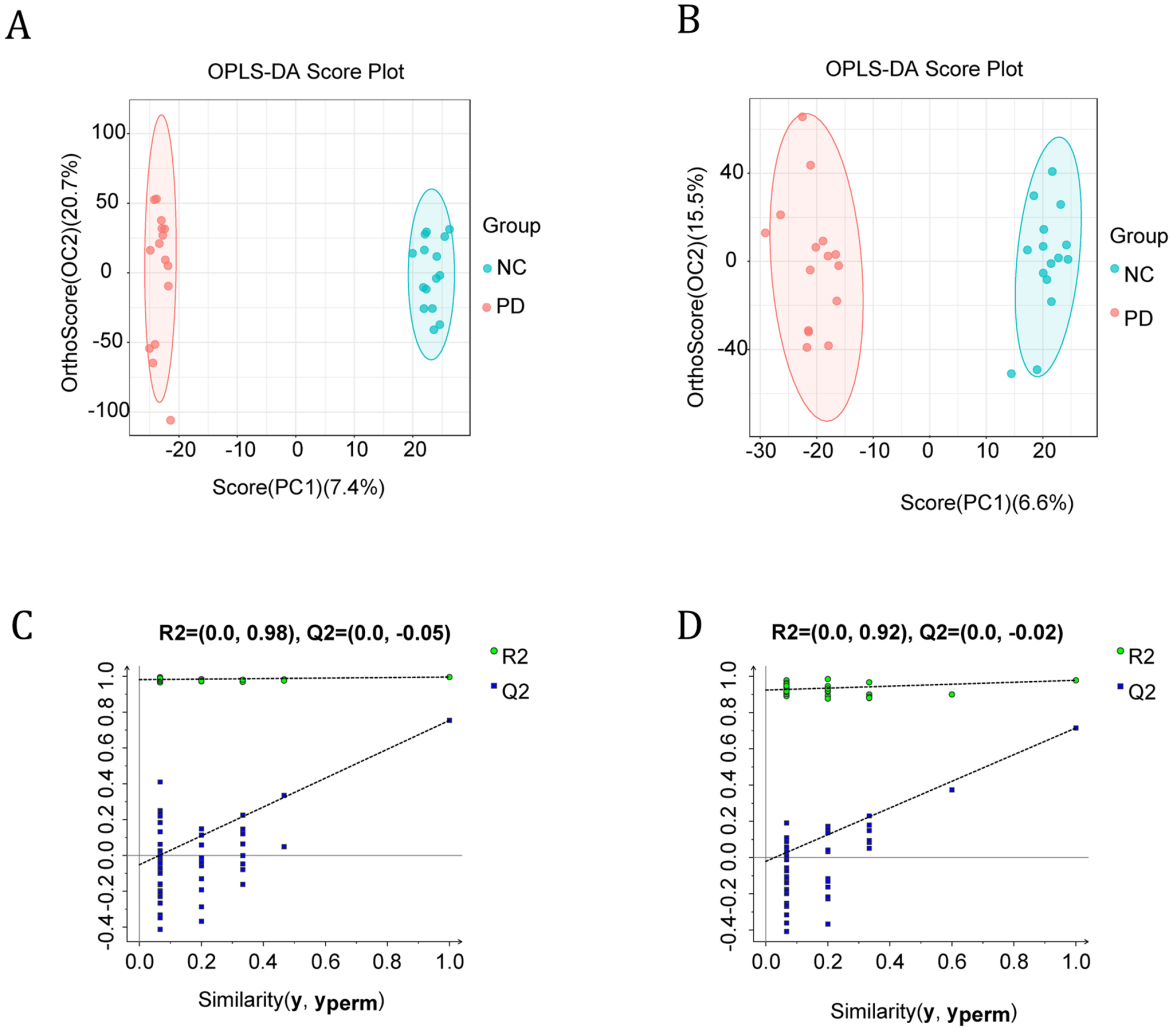
Supervised multivariate analysis and validation of metabolomic differences between PD patients and controls. **(A, B)** OPLS-DA (orthogonal partial least squares discriminant analysis) score plots based on metabolic features in the positive ion mode **(A)** and negative ion mode **(B)**. Clear separation between PD patients and NC indicates distinct metabolic signatures. For the positive mode model (1 + 2 + o), the cumulative parameters were *R*^2^*X* = 0.325, *R*^2^*Y* = 0.996, and *Q*^2^ = 0.754. For the negative mode model (1 + 1 + o), the cumulative parameters were *R*^2^*X* = 0.221, *R*^2^*Y* = 0.978, and *Q*^2^ = 0.715. **(C, D)** Permutation test plots (*n* = 100) for the corresponding OPLS-DA models in the positive **(C)** and negative ion modes **(D)**. In both cases, the original model’s *Q*^2^ value (blue dot) exceeds all permuted *Q*^2^ values, and the regression lines exhibit negative intercepts, supporting model validity and suggesting that the observed separation was unlikely to arise from random permutation alone.

In the positive ion mode, the OPLS-DA model demonstrated strong explanatory and predictive performance, with cumulative parameters of *R*^2^*X* = 0.325, *R*^2^*Y* = 0.996, and *Q*^2^ = 0.754. The model structure comprised 1 predictive and 2 orthogonal components (1 + 2 + o). As shown in Figure 2A, clear separation along the first predictive component (t[1]), which accounted for 7.4% of the total variance, confirmed distinct metabolic profiles between PD patients and NC. Permutation testing (Figure 2C) further validated model robustness: the original *Q*^2^ value (0.754, blue dot) exceeded all 100 permuted *Q*^2^ values (range: −0.05 to 0.45), and the regression line showed a negative intercept with *R*^2^ = 0.95, supporting that the observed class separation was unlikely to arise from random permutation alone.

Similarly, in the negative ion mode, the OPLS-DA model also exhibited strong group discrimination, with cumulative parameters of *R*^2^*X* = 0.221, *R*^2^*Y* = 0.978, and *Q*^2^ = 0.715. The model structure consisted of 1 predictive and 1 orthogonal component (1 + 1 + o). As illustrated in Figure 2B, separation along *t*[1] (explaining 6.6% of variance) indicated consistent metabolic differences independent of ion mode. The 100-fold permutation test (Figure 2D) confirmed the model’s validity: the observed *Q*^2^ (0.715) surpassed all permuted values (range: −0.02 to 0.38), and the regression line demonstrated a negative intercept with *R*^2^ = 0.92, excluding the possibility of model overfitting.

Together, these results demonstrate that the OPLS-DA models derived from both ionization modes are statistically supported and technically reproducible. The high *Q*^2^ values (>0.7) and carefully controlled permutation validation support the robustness of these models for capturing PD-related metabolic disturbances.

Following untargeted LC–MS analysis, a total of 7,558 and 6,891 metabolic features (Table 2) were detected in the positive and negative ion modes, respectively. Subsequent OPLS-DA modeling and differential feature screening using the criteria of VIP > 1.0 and *p* < 0.05 yielded 1,208 and 903 features in the positive and negative ion modes, respectively. Specifically, 671 features were upregulated and 537 were downregulated in positive mode, while 613 were upregulated and 290 were downregulated in negative mode. These preliminary results are summarized in Table 2 and provided the basis for subsequent metabolite annotation and biological interpretation.

**Table 2:** Overview of metabolomics feature processing and differential metabolite identification.

Step	Description	Positive mode	Negative mode	Combined
Raw features detected	LC–MS feature extraction	7,558	6,891	14,449
QC-filtered features	RSD < 30% retained	–	–	High-quality subset
Annotated metabolites	Database + MS/MS annotation	–	–	2,640
Statistical screening	VIP > 1.0 and *p* < 0.05	1,208	903	394 annotated metabolites
Upregulated features	PD vs. NC	671	613	1,284
Downregulated features	PD vs. NC	537	290	827
ML input set	Features used for ML	–	–	394

### 3.4 Identification and annotation of differential metabolites

**Figure 3:**
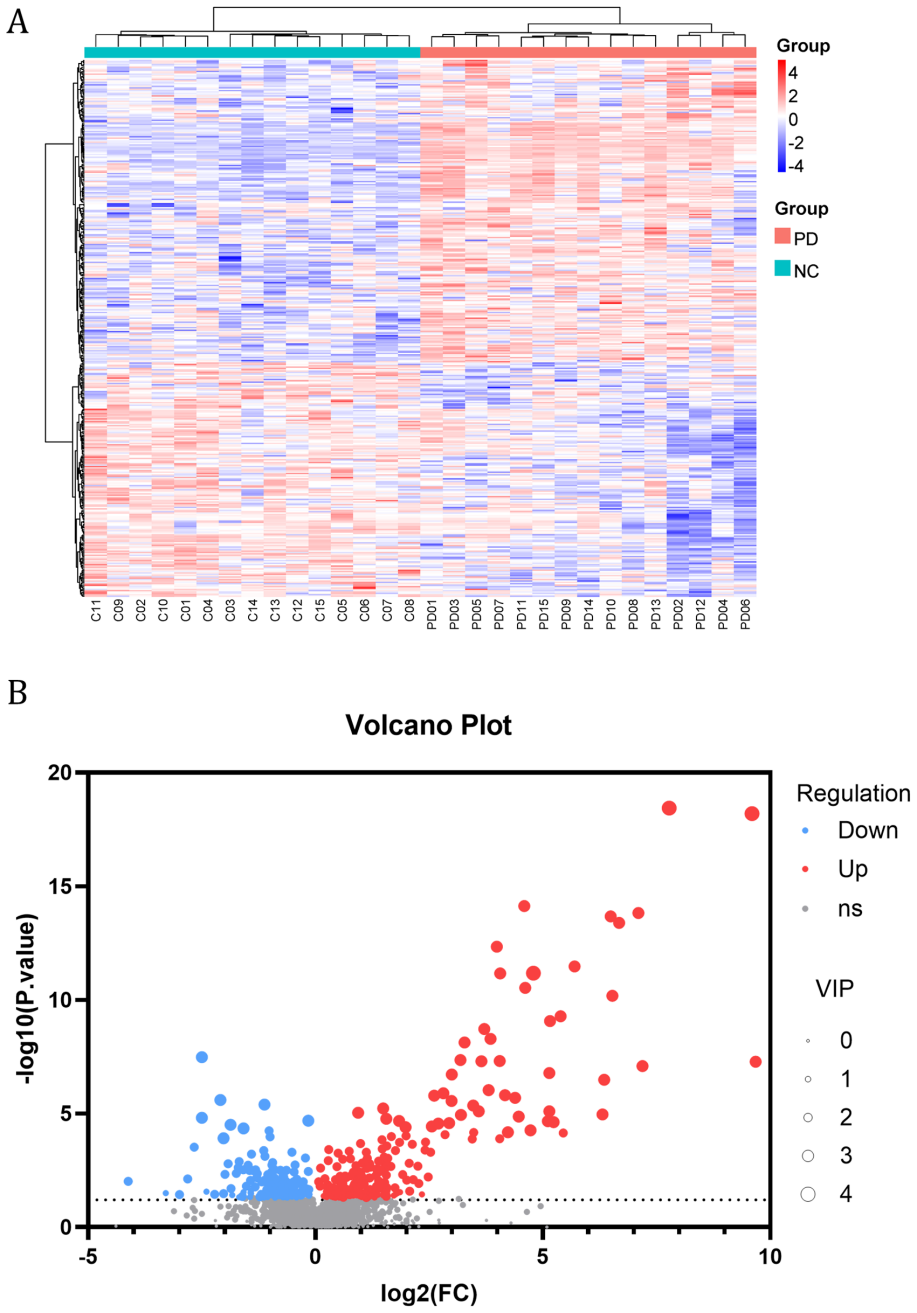
Visualization of differential metabolites between PD patients and NC. **(A)** Hierarchical clustering heatmap of differential metabolites between PD patients and NC. The heatmap displays 394 candidate differential metabolites with VIP > 1.0 and *p* < 0.05, based on MS/MS-annotated features. Rows represent metabolites, and columns represent individual urine samples (PD group on the right, NC group on the left). *Z*-score normalization was applied across samples, and both rows and columns were hierarchically clustered. Red indicates high relative abundance, and blue indicates low abundance. The clustering dendrograms reveal clear separation between PD and NC groups, with PD samples forming subclusters suggestive of metabolic heterogeneity. Distinct metabolite modules were also observed, including clusters of metabolites upregulated in PD (top-right) and clusters showing lower expression in PD (bottom-left). **(B)** Volcano plot of differential metabolites between PD patients and NC. Each point represents a metabolite annotated by MS/MS. The *x*-axis shows log₂ fold change (PD vs. NC), and the *y*-axis shows –log_10_(*p*-value). Metabolites meeting the exploratory screening criteria (VIP > 1.0 and raw *p* < 0.05) are highlighted. Benjamini–Hochberg FDR correction was additionally applied during statistical evaluation: red dots indicate significantly upregulated features in PD, blue dots indicate significantly downregulated features, and gray dots represent non-significant features. Dot size corresponds to VIP value, with larger points indicating greater discriminative power.

Among the statistically screened features, 394 metabolites could be confidently annotated based on MS/MS-supported database matching and were therefore retained for downstream interpretation and machine learning analysis. As shown in the hierarchical clustering heatmap (Figure 3A), samples were clearly separated into PD and NC groups, with PD samples forming 2–3 distinct subclusters. This clustering structure indicates a high degree of metabolic heterogeneity within the PD group. On the metabolite axis, several co-expression clusters were observed. Notably, a group of metabolites in the bottom-left region of the heatmap exhibited coordinated upregulation in PD samples, while a top-right cluster showed consistent downregulation, suggesting a consistent suppression pattern of several metabolites in PD.

The volcano plot (Figure 3B) further illustrated the distribution of differential metabolites based on log2 fold change and statistical significance. Raw *p*-values are displayed for visualization purposes, while Benjamini–Hochberg FDR correction was additionally applied during statistical evaluation. Although no strict fold-change cutoff was applied, many candidate metabolites exhibited substantial fold differences in addition to statistical significance (Supplementary Table S1). A considerable number of features showed strong statistical significance and large fold changes, including 223 upregulated and 171 downregulated metabolites. The use of VIP as dot size highlights key variables with high classification relevance.

### 3.5 Machine learning-based feature selection

To refine and prioritize candidate biomarkers from the pool of MS/MS-confirmed differential metabolites, three supervised machine learning models were applied: RF, SVM, and PLS-DA. Each method adopts a distinct variable selection strategy to assess the classification relevance of metabolites.

**Figure 4:**
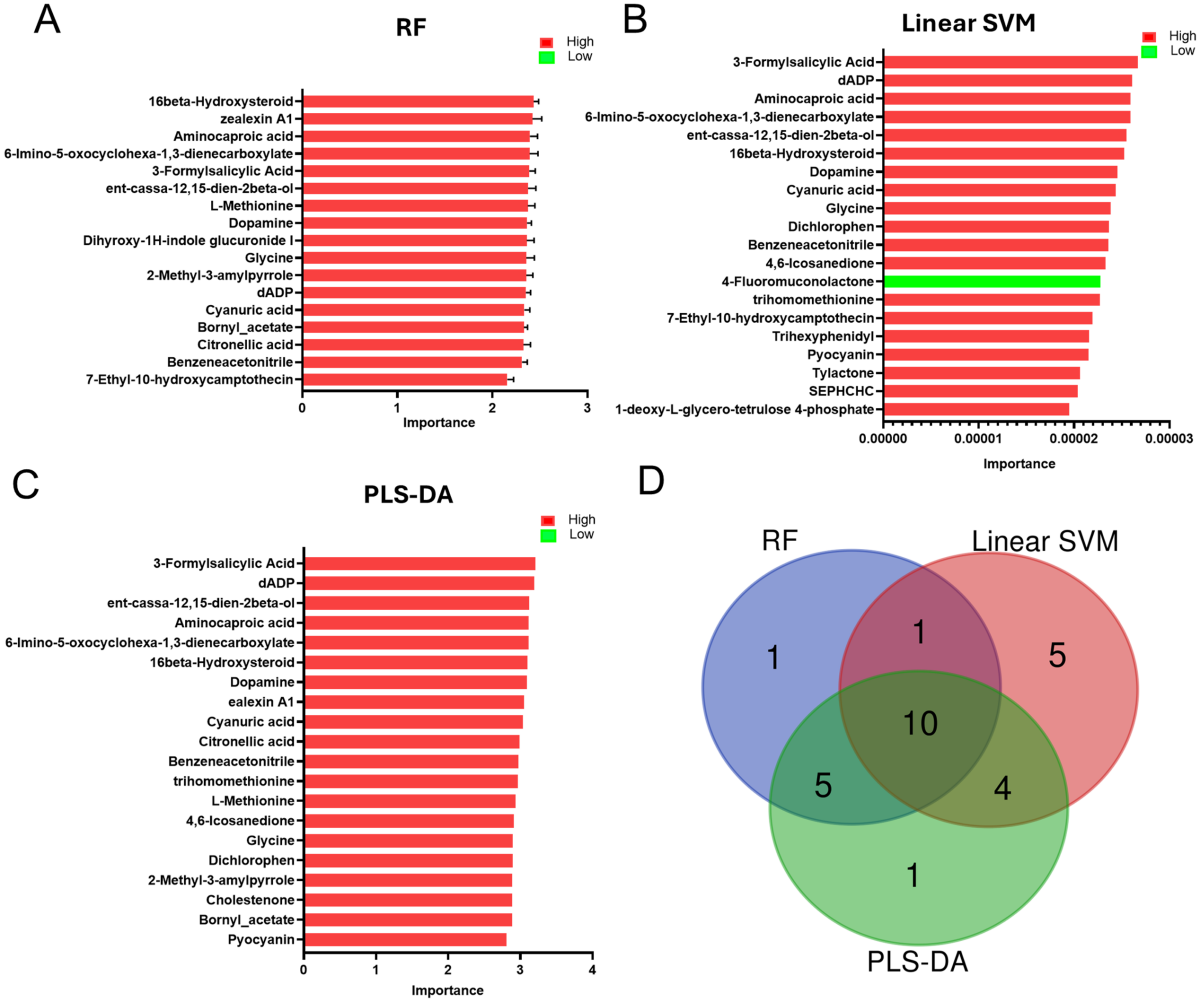
Machine learning-based biomarker selection using RF, SVM, and PLS-DA. **(A)** RF analysis: Ten independent RF models identified top-ranked metabolic features based on mean decrease accuracy. The RF-consensus set comprises features consistently ranked in the top 20 across all runs. Features are ordered top to bottom by decreasing importance. Red indicates up-regulation, green indicates down-regulation. **(B)** SVM analysis: Linear SVM with RFE ranked features by weight coefficients; the top 20 metabolites were retained. Features are ordered top to bottom by decreasing importance. Red indicates up-regulation, green indicates down-regulation. **(C)** Partial least squares discriminant analysis (PLS-DA): Features were prioritized using VIP scores, with the top 20 metabolites selected. Features are ordered top to bottom by decreasing importance. Red indicates up-regulation, green indicates down-regulation. **(D)** Venn diagram illustrating overlaps among the top 20 biomarker candidates identified by RF, SVM, and PLS-DA.

For RF modeling, ten independent models were trained using bootstrapped subsets of the dataset. Metabolites were ranked in each model based on the Mean Decrease Accuracy (MDA), which reflects the contribution of each variable to model accuracy. A set of metabolites consistently ranked in the top 20 across all ten RF runs was defined as the RF consensus signature, highlighting features with high stability and predictive value (Figure 4A).

SVM modeling was performed using a linear kernel combined with RFE. Metabolites were ranked based on the absolute magnitude of the weight coefficients, and the top 20 features with the highest discriminative power were retained (Figure 4B).

For PLS-DA, metabolites were ranked according to their VIP scores. The top 20 features with VIP > 1.5 were selected as potential discriminants contributing most strongly to group separation between PD and control samples (Figure 4C).

As shown in the Venn diagram (Figure 4D, Table 3), 10 metabolites were commonly identified by all three models, constituting a robust consensus candidate set for downstream refinement. These overlapping metabolites reflect high model-agnostic relevance and stability, offering strong potential as diagnostic or mechanistic indicators of PD.

**Table 3:** Overlap of candidate metabolites identified by RF, SVM, and PLS-DA.

Overlap group	Number	Metabolite names
Linear SVM ∩ PLS-DA ∩ RF	10	Dopamine
		Benzeneacetonitrile
		Cyanuric acid
		3-Formylsalicylic Acid
		6-Imino-5-oxocyclohexa-1,3-dienecarboxylate
		16beta-Hydroxysteroid
		Glycine
		Aminocaproic acid
		dADP
		ent-cassa-12,15-dien-2beta-ol
Linear SVM ∩ RF	1	7-Ethyl-10-hydroxycamptothecin
PLS-DA RF	5	zealexin A1
		L-Methionine
		2-Methyl-3-amylpyrrole
		Bornyl_acetate
		Citronellic acid
Linear SVM PLS-DA	4	Dichlorophen
		trihomomethionine
		Pyocyanin
		4,6-Icosanedione
RF	1	Dihyroxy-1H-indole glucuronide I
Linear SVM	5	Trihexyphenidyl
		1-deoxy-L-glycero-tetrulose 4-phosphate
		SEPHCHC
		Tylactone
		4-Fluoromuconolactone
PLS-DA	1	Cholestenone

This multi-model selection strategy improves confidence in exploratory candidate prioritization by integrating multiple perspectives on feature importance. However, external validation remains necessary to establish generalizability.

### 3.6 Candidate biomarker assessment to multivariate model construction

#### 3.6.1 Univariate evaluation of candidate biomarkers

**Figure 5:**
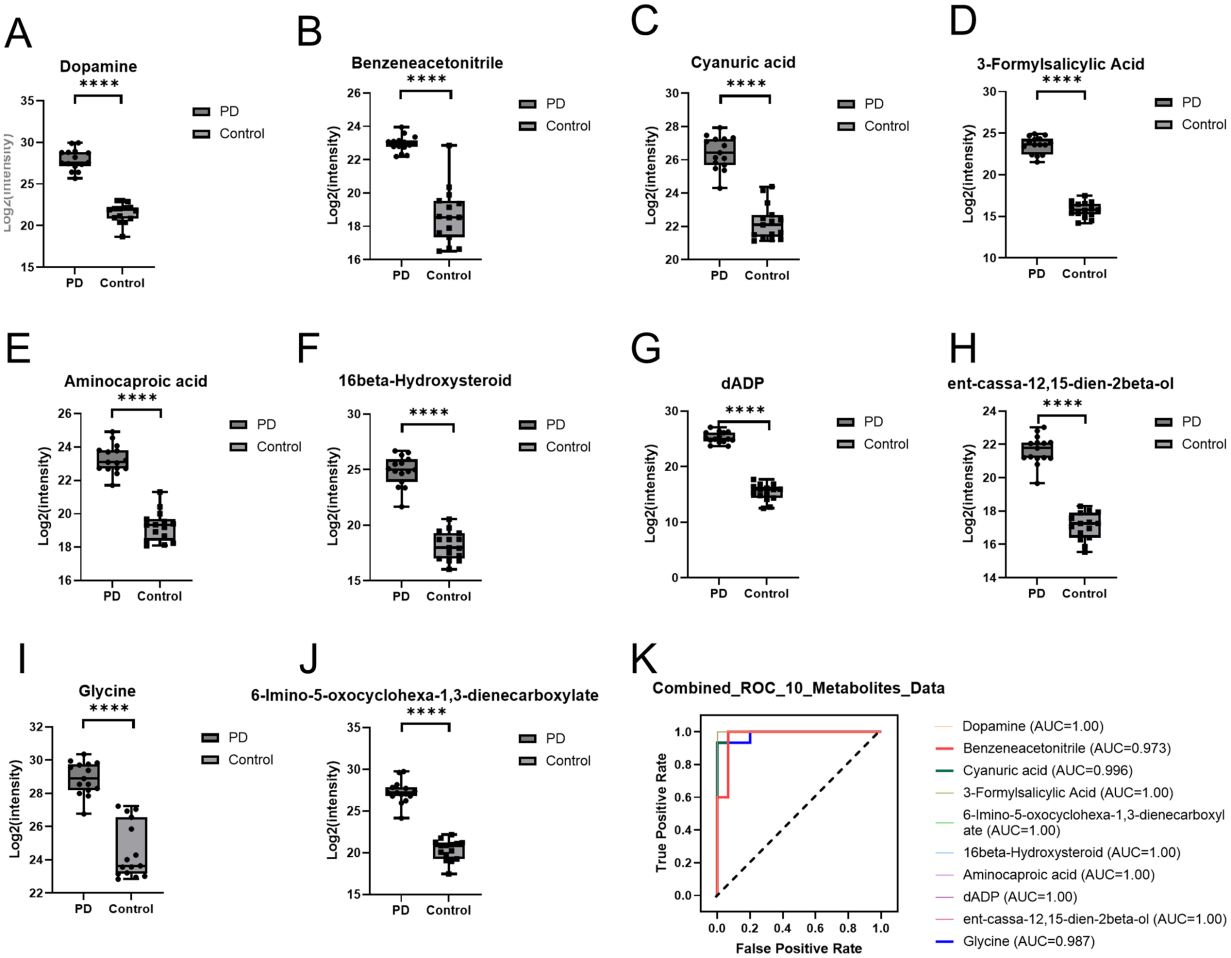
Univariate evaluation of candidate metabolites. **(A–J)** Boxplots showing the relative abundance of ten machine learning–selected metabolites between PD patients and NC. All selected metabolites demonstrated consistent directional changes and statistical separation between groups. **(K)** ROC curves of individual metabolites. Each curve represents one metabolite’s performance in distinguishing PD from control samples. All ten metabolites yielded an AUC of ≥0.97, with seven metabolites achieved an apparent AUC of 1.00 within this exploratory cohort, which likely reflects the highly controlled matched design together with the limited sample size.

To evaluate the diagnostic performance of each selected metabolite individually, receiver operating characteristic (ROC) analysis was conducted based on their quantitative profiles in PD and control groups. As shown in Figures 5A–J, all ten candidate biomarkers exhibited clear and statistically significant abundance differences between groups, with most showing minimal to no overlap.

Corresponding ROC curves (Figure 5K) confirmed the high classification potential of these metabolites. Notably, seven out of ten showed complete separation within this exploratory cohort (AUC = 1.00), while the remaining three metabolites also performed strongly with AUC values exceeding 0.97. These results suggest that even as standalone features, these metabolites offer excellent predictive power for distinguishing PD patients from controls.

#### 3.6.2 Feature selection and collinearity elimination prior to multivariate modeling

**Figure 6:**
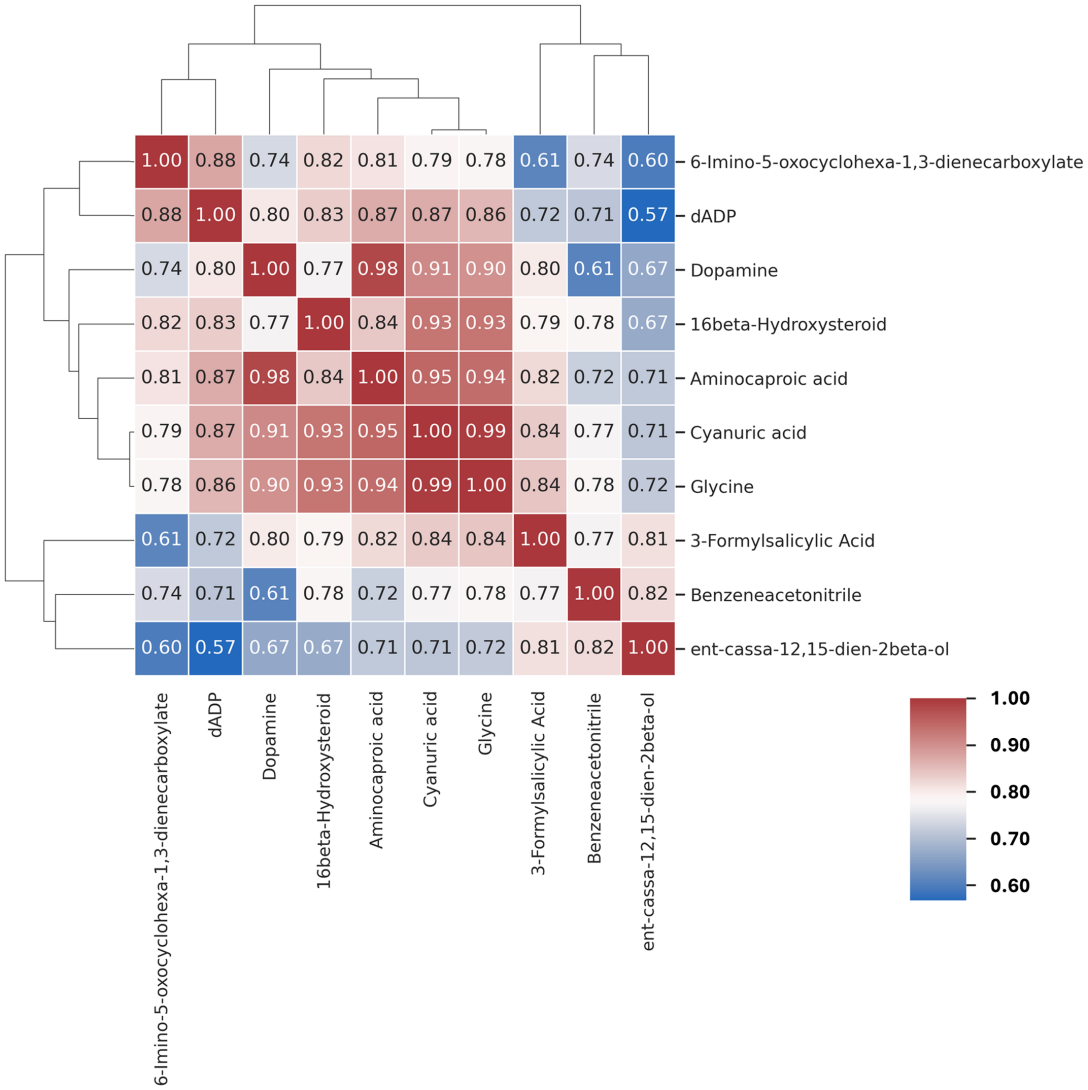
Correlation heatmap of selected Parkinson’s disease-related metabolites. This heatmap illustrates the pairwise Pearson correlation coefficients among 10 significantly altered urinary metabolites associated with Parkinson’s disease (PD). Metabolites were selected based on their discriminative power and biological relevance, including dopamine, cyanuric acid, and 3-formylsalicylic acid. The correlation matrix is color-coded, with red indicating strong positive correlations (*r* approaching 1.0) and blue indicating weaker correlations (*r* approaching 0.6). Hierarchical clustering was applied to both rows and columns to reveal co-regulated metabolite modules.

To ensure relatively stable and interpretable classification models, we implemented a structured variable refinement process before multivariate model construction. Candidate biomarkers identified by machine learning models were further filtered based on pharmacological relevance, multicollinearity, and inter-metabolite correlation (Figure 6).

First, Dopamine was excluded due to its direct association with L-DOPA metabolism and known sensitivity to PD medications. Glycine and 16β-Hydroxysteroid were flagged as potentially drug-sensitive due to their known involvement in neurotransmitter and steroid metabolism.

Second, variance inflation factors (VIFs) were calculated for each candidate. Variables with VIF > 10 were iteratively removed to avoid multicollinearity. Among them, Aminocaproic acid and Glycine showed extremely high VIFs and strong pairwise correlation, and were excluded. Although Cyanuric acid exhibited a high VIF, it was retained as the representative metabolite within a highly correlated cluster due to its stronger statistical significance and biological interpretability relative to correlated candidates.

Third, we calculated Pearson correlation coefficients for all pairs of metabolites. As shown in Figure 6, several candidates (e.g., Dopamine, Aminocaproic acid, Glycine) formed highly correlated clusters, supporting their removal.

After refinement, six metabolites remained as non-redundant candidate variables. Among them, five metabolites were selected to construct the final minimal classification panel: Cyanuric acid, Benzeneacetonitrile, 3-Formylsalicylic Acid, dADP, and ent-cassa-12,15-dien-2beta-ol (Table 4).

**Table 4:** Filtering of candidate biomarkers based on drug interference, VIF, and correlation.

Metabolite	VIF	Pharmacological relevance	Highly correlated (*r* > 0.9)	Inclusion status
Aminocaproic acid	248.90	Possible	Cyanuric acid, glycine	Excluded
Dopamine	198.61	Yes	Cyanuric acid, glycine	Excluded
Cyanuric acid	130.51	No	Glycine, aminocaproic acid	Included
Glycine	98.52	Possible	Cyanuric acid, aminocaproic acid	Excluded
Benzeneacetonitrile	18.38	No	Moderate correlation	Included
16β-hydroxysteroid	18.30	Possible	Cyanuric acid	Excluded
dADP	11.64	No	None	Included
3-formylsalicylic acid	10.77	No	None	Included
6-Imino-5-oxocyclohexa-1,3-dienecarboxylate	9.97	No	None	Included (Retained but not included in final minimal panel)
ent-cassa-12,15-dien-2beta-ol	5.03	No	None	Included

Detailed statistical and metabolomic characteristics of the final prioritized metabolite panel, including retention time, fold change, *p*-value, FDR, ionization mode, and original OPLS-DA VIP values, are summarized in Table 5. These parameters provide a consolidated overview of the refined exploratory biomarker candidates selected through the sequential machine learning consensus and feature refinement workflow.

**Table 5:** Summary of the final exploratory urinary metabolite panel following machine learning consensus selection and feature refinement.

Metabolite	m/z	RT	FC	log2FC	*p*-value	FDR	VIP	Pos/neg	Selection stage
Cyanuric acid	168.1133	55.3	16.70	4.06	6.93 × 10^−12^	1.94 × 10^−9^	3.31	pos	ML consensus + final panel
Benzeneacetonitrile	233.1053	72.0	9.16	3.19	4.46 × 10^−8^	7.70 × 10^−6^	3.31	neg	ML consensus + final panel
3-formylsalicylic acid	149.0961	271.7	219.44	7.78	3.66 × 10^−19^	2.45 × 10^−15^	3.57	pos	ML consensus + final panel
dADP	368.0552	201.4	774.39	9.6	6.48 × 10^−19^	2.45 × 10^−15^	3.56	neg	ML consensus + final panel
ent-cassa-12,15-dien-2beta-ol	272.2222	329.2	24.03	4.59	7.42 × 10^−15^	7.01 × 10^−12^	3.46	pos	ML consensus + final panel

Metabolites shown in this table represent the final exploratory metabolite panel obtained after consensus selection across RF, SVM, and PLS-DA models, followed by pharmacological relevance assessment and collinearity filtering. VIP values were derived from the initial OPLS-DA differential feature screening stage prior to machine learning-based consensus selection and reflect contribution to global group discrimination rather than variable importance within the final refined classification models.

#### 3.6.3 Multivariate model evaluation

**Figure 7:**
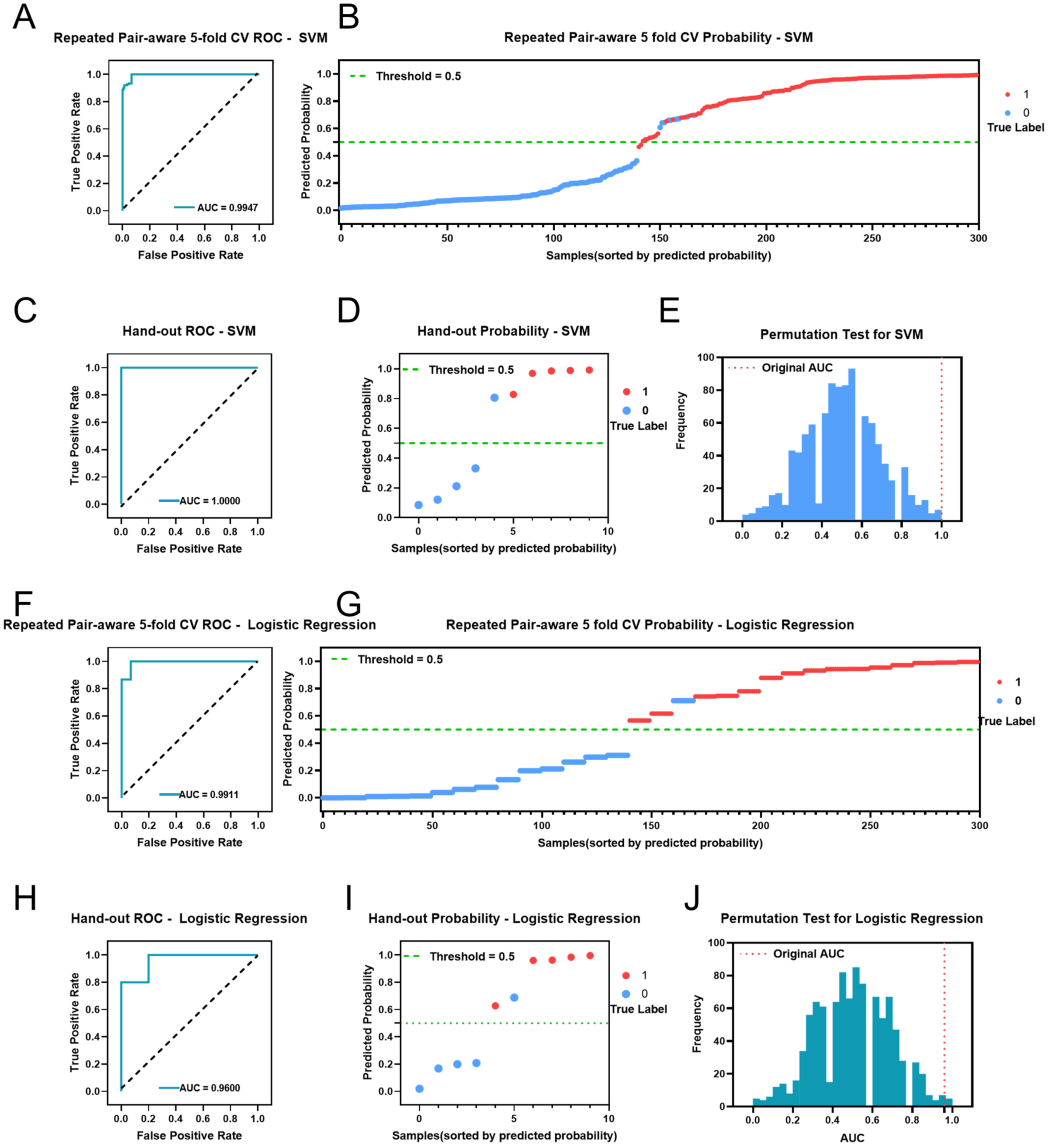
Performance evaluation of multivariate classification models using selected metabolite panel. **(A–E)** Results from the SVM classifier: **(A)** Aggregated ROC curve based on repeated 5-fold pair-aware cross-validation. **(B)** Predicted probability distribution across PD and control samples in CV. **(C)** ROC curve on independent hold-out validation set. **(D)** Predicted probability distribution on hold-out set. **(E)** Permutation test of AUC on hold-out data (1,000 iterations), red dashed line indicates observed AUC. **(F–J)** Results from the logistic regression (LR) classifier: **(F)** Aggregated ROC curve based on repeated 5-fold pair-aware cross-validation. **(G)** Predicted probability distribution across PD and control samples in CV. **(H)** ROC curve on independent hold-out validation set. **(I)** Predicted probability distribution on hold-out set. **(J)** Permutation test of AUC on hold-out data (1,000 iterations), red dashed line indicates observed AUC.

To assess the diagnostic performance of the final metabolite panel, multivariate classification models were built using SVM and Logistic Regression (LR) algorithms. Both models were trained using a repeated pair-aware 5-fold cross-validation strategy and evaluated on an independent hold-out set, with results summarized in Figure 7.

In the SVM model, the cross-validated ROC curve (Figure 7A) demonstrated excellent discriminative ability, with high AUC values across folds. The predicted probabilities (Figure 7B) showed clear separation between PD patients and controls. On the hold-out set, the ROC curve (Figure 7C) remained consistent with cross-validation, demonstrating consistent performance within the internal hold-out evaluation. The predicted probability plot (Figure 7D) again indicated strong group separation. Permutation testing (Figure 7E) validated the significance of model performance, with the observed AUC significantly exceeding the null distribution (*p* < 0.001).

Similarly, the Logistic Regression model (Figures 7F–J) exhibited strong classification performance. ROC curves for both CV and hold-out sets (Figures 7F,H) showed strong agreement. Probability distributions (Figures 7G,I) revealed well-separated groups. The permutation test (Figure 7J) further confirmed statistical significance of model performance (*p* < 0.001).

Together, these findings indicate that both SVM and Logistic Regression models trained on the selected metabolite panel achieved strong discrimination within this matched exploratory cohort. However, because the study was conducted in a limited internal dataset, these findings should be interpreted as preliminary and hypothesis-generating rather than evidence of established clinical generalizability.

## 4 Discussion

In this exploratory pilot study, we employed a rigorous matched patient-spouse design combined with untargeted metabolomics and multi-model machine learning to investigate urinary metabolic alterations in PD. The primary objective was not to establish a validated diagnostic tool, but to utilize this highly controlled approach as a discovery platform for identifying high-priority candidate biomarkers that merit future large-scale investigation. We acknowledge at the outset that the sample size (*n* = 15 pairs), while a direct consequence of the stringent and logistically challenging paired design, represents the key limitation of this work and necessitates that all findings be interpreted as preliminary and hypothesis-generating.

### 4.1 The value and challenge of a matched-pair design for metabolic discovery

Our OPLS-DA models demonstrated clear metabolic separation between groups, consistent with systemic metabolic effects in PD observable in peripheral biofluids (8, 9). Crucially, this separation was achieved within a cohort meticulously matched for lifelong environmental and lifestyle confounders. This design, though inherently limiting sample size due to the scarcity of eligible elderly patient-spouse pairs, significantly strengthens the inference that the observed differences are more likely linked to PD pathology rather than extraneous factors. The observed metabolic heterogeneity within the PD group (10) may thus reflect truer biological variation related to disease subtype or progression, rather than noise from uncontrolled confounders. Such heterogeneity may also reflect differences in disease duration, symptom severity, or medication exposure among patients. We acknowledge that the spouse-paired design also introduced an inherent sex imbalance and sex-specific metabolic effects therefore cannot be fully excluded between groups, which may contribute to part of the observed metabolic variation and should be addressed in future larger cohorts with stratified analyses.

### 4.2 A consensus machine learning strategy for prioritizing candidates

To navigate the high-dimensional data from our unique but small cohort, we applied a consensus feature selection strategy across multiple ML algorithms (11–13). This approach aimed to enhance the robustness of candidate identification in a discovery setting. The intersection of models yielded ten consensus candidates, and subsequent filtering for pharmacological relevance and collinearity refined this to a preliminary five-metabolite panel. We emphasize that the outstanding internal validation performance (AUC > 0.95) of models built on this panel, while encouraging, must be viewed in the context of the same dataset used for discovery and the limited sample size. It primarily indicates a strong, consistent signal within this specific, controlled cohort rather than proven generalizability. In addition, because biomarker prioritization was performed prior to classifier evaluation, some degree of optimistic bias cannot be excluded. The occurrence of complete or near-complete discrimination for several metabolites likely reflects both the highly controlled matched design and the limited sample size, and should not be interpreted as evidence of immediate clinical applicability. Therefore, the current classification performance should be interpreted cautiously as exploratory internal validation.

### 4.3 Biological plausibility of preliminary candidates

The identified metabolites point to several biologically plausible pathways potentially associated with PD pathophysiology, including altered xenobiotic metabolism, mitochondrial dysfunction, and gut–microbiota-related metabolic disturbances. Metabolites such as dADP, which is involved in nucleotide metabolism (14), together with compounds linked to microbial or dietary metabolism [e.g., 3-Formylsalicylic Acid (15) and Cyanuric acid (16)], suggest systemic metabolic alterations extending beyond the central nervous system. These observations are consistent with emerging concepts in PD research emphasizing mitochondrial impairment, oxidative stress, and gut–brain axis involvement as major contributors to disease progression (17, 18).

Notably, the elevated urinary levels of Cyanuric acid (27) and Benzeneacetonitrile in PD patients—despite substantial shared environmental and dietary backgrounds with spousal controls—may reflect altered xenobiotic handling or detoxification capacity rather than simple differences in environmental exposure alone. Liuzhou, as a major industrial hub with dense automotive manufacturing and heavy machinery production, retains substantial long-term exposure potential to traffic-related pollutants (e.g., NOx, VOCs) and industrial volatile compounds (19). Despite significant ecological improvements in recent years, these persistent industrial- and traffic-derived xenobiotics may still contribute to the chronic environmental toxicant burden and interact with host metabolic susceptibility in PD, although direct exposure measurements were not performed in this study.

Environmental toxicants, including industrial solvents and pesticides, have been repeatedly implicated as risk factors for PD through mechanisms involving oxidative stress and mitochondrial injury (16). Although the direct neurobiological relevance of these specific metabolites remains unclear, their accumulation may suggest altered systemic detoxification or metabolic processing capacity in PD patients.

The increased abundance of dADP further supports the concept of impaired energy metabolism in PD. Recent targeted metabolomics studies have demonstrated significant disruptions in purine metabolism and ATP recycling pathways in PD patients, linking nucleotide metabolic dysfunction to impaired neuronal survival and mitochondrial stress (20). In parallel, the elevation of 3-Formylsalicylic Acid, a metabolite associated with aromatic compound metabolism, is consistent with previous evidence indicating that aromatic amino acid metabolic pathways are altered in PD and may be influenced by gut microbiota dysbiosis (21, 22). These findings align with growing evidence that systemic metabolic abnormalities in PD extend beyond the brain and involve broader host–microbiome metabolic interactions.

The detection of ent-cassa-12,15-dien-2beta-ol, a diterpenoid metabolite derived from rice (*Oryza sativa*) phytoalexin biosynthesis (23), provides a distinctive lens into diet-microbiota-host interactions within this Liuzhou cohort. Given that both PD patients and their spousal controls share a comparable rice-based dietary background (e.g., Luosifen consumption), the differential urinary abundance of this plant-derived metabolite is unlikely driven by dietary intake alone. Rather, it may reflect increased intestinal permeability and altered gut microbial metabolism, thereby permitting the abnormal translocation of dietary diterpenoids into the systemic circulation (21, 24). This finding aligns with the broader evidence that gut barrier dysfunction and microbiota-derived signaling are integral to PD pathogenesis via the gut-brain axis (25), suggesting that regionally specific dietary metabolites can serve as sensitive probes for detecting host physiological impairments in a paired-cohort setting (29).

Together, these connections provide a plausible biological context for the identified metabolites and support their prioritization for further investigation, although they do not constitute mechanistic evidence.

Notably, While Dopamine showed near-complete discrimination between groups, recent large-scale metabolomics studies confirm that such alterations are predominantly driven by dopaminergic medication (e.g., L-DOPA therapy) rather than disease status itself (26), necessitating the exclusion of treatment-confounded metabolites from the diagnostic panel. Nevertheless, because these metabolite annotations were derived from untargeted LC–MS/MS database matching rather than authentic chemical standards, the biological interpretation of several compounds should remain cautious pending targeted validation.

### 4.4 Conclusion and future directions: from pilot discovery to validation

Because all PD patients were under stable dopaminergic medication, we cannot entirely exclude subtle drug effects on the identified metabolites (28). Detailed levodopa equivalent daily dose (LEDD) information was not available for all participants and therefore could not be incorporated into stratified metabolite analyses. Future validation in drug-naïve cohorts is warranted. The principal contribution of this pilot study is twofold. First, it demonstrates the utility of a matched patient-spouse design as a powerful, albeit sample-size-limiting, strategy for generating robust candidate biomarker signals in metabolomic studies of aging-related diseases. Second, it proposes a specific set of urinary metabolites (Cyanuric acid, Benzeneacetonitrile, 3-Formylsalicylic Acid, dADP, and ent-cassa-12,15-dien-2beta-ol) as high-priority targets for independent validation.

Consequently, the essential next step is external validation. Future studies should assess the performance of this panel in larger, independent, and more heterogeneous cohorts, including drug-naïve patients, while implementing nested cross-validation frameworks and targeted quantitative validation to further minimize feature-selection bias and improve generalizability assessment. Furthermore, integrating these metabolic findings with multi-omics data (e.g., microbiome, transcriptomics) will be critical to elucidate their functional relevance and mechanistic role in PD pathogenesis.

## Data Availability

The raw data supporting the conclusions of this article will be made available by the authors, without undue reservation.

## Supplementary material

SUPPLEMENTARY TABLE S1 Complete differential metabolite dataset with annotation and statistical metrics.
